# Shear Stress Targeted Delivery of Nitroglycerin to Brain Collaterals Improves Ischaemic Stroke Outcome

**DOI:** 10.1002/advs.202506276

**Published:** 2025-08-13

**Authors:** Magdalena Litman, Sara Azarpeykan, Rebecca J Hood, Kristy Martin, Debbie Pepperall, Daniel Omileke, Fern Williams, Oktay Uzun, Deen Bhatta, Yuen K Yong, Alex Chan, Nicholas Hough, Sarah Johnson, Pablo Garcia Bermejo, Ferdinand Miteff, Carlos Garcia Esperon, Yvonne Couch, Alastair M Buchan, Neil J Spratt, Netanel Korin, Donald E Ingber, Daniel J Beard

**Affiliations:** ^1^ School of Biomedical Science and Pharmacy The University of Newcastle Newcastle 2308 Australia; ^2^ Discipline of Anatomy and Pathology School of Biomedicine Faculty of Health and Medical Sciences The University of Adelaide Adelaide 5000 Australia; ^3^ Heart and Stroke Programme Hunter Medical Research Institute Newcastle 2305 Australia; ^4^ Wyss Institute of Biologically Inspired Engineering Harvard University Boston MA 02215 USA; ^5^ School of Engineering University of Newcastle Callaghan NSW 2308 Australia; ^6^ Department of Neurology John Hunter Hospital Hunter New England Local Health District Newcastle 2305 Australia; ^7^ Acute Stroke Programme Radcliffe Department of Medicine University of Oxford Oxford OX39DU UK; ^8^ Department of Biomedical Engineering Technion‐Israel Institute of Technology Haifa 3200003 Israel; ^9^ Vascular Biology Program and Department of Surgery Children's Hospital Boston and Harvard Medical School Boston MA 02115 USA; ^10^ Harvard John A. Paulson School of Engineering and Applied Sciences Cambridge MA 02134 USA

**Keywords:** collateral therapeutics, collaterals, ischaemic stroke, nanotherapeutics, shear stress

## Abstract

In patients with ischaemic stroke, retrograde perfusion of the penumbra by leptomeningeal collateral vessels (LMCs) strongly predicts clinical outcome, suggesting that enhancing LMC flow can offer a novel therapeutic approach. Using in vivo measurements and computational modelling it is shown that LMCs experience elevated fluid shear stress that is significantly higher than that in other blood vessels during ischaemic stroke in rats and humans. We exploit this to selectively enhance flow in LMCs using shear‐activated nanoparticle aggregates carrying the vasodilator nitroglycerin (NG‐NPAs) that specifically release drug in regions of vessels with high wall shear stress (≥100 dyne cm^−2^). NG‐NPAs significantly increased LMC‐mediated penumbral perfusion, decreased infarct volume, and reduced neurological deficit without altering systemic blood pressure in a rat ischaemic stroke model. NG‐NPAs also avoided common side effects of systemic nitrate administration, such as systemic hypotension, cerebral vascular steal, cortical vein dilation, or intracranial pressure elevation. Systemic administration of free NG at the maximal tolerated dose, which is ten times higher than the dose of NG used in the NG‐NPAs, do not enhance LMC perfusion and dropped blood pressure. Thus, packaging NG within shear‐activated NPAs can potentially enable this widely available vasodilator to become a highly effective therapeutic for ischaemic stroke.

## Graphical abstract

Shear‐activated nanoparticles carrying nitroglycerin selectively target high‐shear stress collateral vessels during ischaemic stroke, enhancing blood flow to at‐risk brain without systemic side effects. This novel approach significantly improves outcomes in animal models, outperforming conventional nitroglycerin delivery. The findings suggest a promising therapeutic strategy that repurposes a common vasodilator for precise, stroke‐specific intervention.

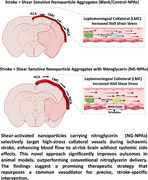

## 1 Introduction

Stroke, resulting in disruption of blood flow to the brain, is the third leading cause of death and a major cause of long‐term disability worldwide.^[^ ^1^ ^]^ Ischaemic stroke, which accounts for 71% of all strokes globally, is caused by cerebral arterial occlusion.^[^ ^2^ ^]^ The goal of acute ischaemic stroke therapies is to salvage the ischaemic penumbra, which is the region of brain tissue beyond the occlusion that is functionally compromised but remains potentially salvageable. The penumbra receives retrograde blood flow via cerebral collateral vessels in the brain's leptomeningeal collateral (LMC) circulation that are formed through end‐to‐end anastomoses of distal branches of adjacent arterial territories.^[^ ^3^ ^]^ The LMCs provide residual retrograde cerebral blood flow when conventional routes of arterial supply are compromised.^[^ ^4^ ^]^

Importantly, advanced clinical imaging over the past decade has led to recognition of the key importance of LMCs in ischaemic stroke outcome.^[^ ^5^ ^]^ The presence of good LMC perfusion is associated with a larger penumbra, better rates of reperfusion, and improved functional outcomes following both thrombolysis and thrombectomy.^[^ ^6^, ^7^, ^8^ ^]^ This is most likely because patients with better collaterals have a large volume of penumbra when they arrive at the hospital.^[^ ^9^, ^10^ ^]^ LMCs have such a large influence on response to reperfusion therapies and stroke outcome that LMC status and penumbral volume are now used to select patients most likely to benefit from reperfusion therapies.^[^ ^11^, ^12^ ^]^ However, a large portion of stroke patients have co‐morbidities such as hypertension (in ≈55% of stroke patients), which is associated with poor LMC status. These patients may therefore benefit greatly from therapeutic interventions that enhance LMC blood flow.^[^ ^13^ ^]^ While the potential benefits of therapeutic approaches that enhance collateral flow and thereby improve stroke outcome are widely recognised, finding an effective method to enhance collateral flow has proved to be very challenging.^[^ ^14^ ^]^

One of the greatest obstacles to developing collateral therapies is the inability to selectively vasodilate LMCs without causing systemic vasodilation and hypotension.^[^ ^15^ ^]^ Healthy cerebral vasculature can autoregulate (vasodilate) in response to low blood pressure and maintain normal perfusion.^[^ ^16^ ^]^ However, arterioles in the ischaemic penumbra may have already reached their limits of autoregulation, and so blood flow becomes entirely dependent on cerebral perfusion pressure.^[^ ^17^ ^]^ Thus, systemic vasodilators that lower blood pressure may have a “reverse Robin Hood” effect – redirecting or stealing blood flow away from the ischaemic brain territory.^[^ ^18^ ^]^ Further, systemic vasodilators have a higher affinity for cerebral veins, leading to venodilation and increased intracranial pressure (ICP, which further reduces cerebral perfusion pressure.^[^ ^15^ ^]^ As a result, clinical trials of systemic vasodilators in stroke showed no change in penumbral blood flow^[^ ^19^ ^]^ and no improvement in patient outcome.^[^ ^20^ ^]^ Based on this evidence, the American Heart Association Guidelines advise against the use of systemic vasodilatory agents for ischaemic stroke treatment.^[^ ^21^ ^]^

This present study is based on the hypothesis that selective dilation of LMCs may be achieved by targeting vasodilators to these vessels, based on work which has revealed that LMCs have unique blood flow haemodynamics that are potentially exploitable therapeutically.^[^ ^22^ ^]^ More specifically, when the middle cerebral artery (MCA) is occluded in rats, the large pressure differential between the occluded and patent vascular territories causes a 10‐fold increase in blood flow velocity through LMCs and an associated increase in wall shear stress (WSS) (>100 dyne cm^−2^) selectively within these vessels.^[^ ^23^, ^24^ ^]^ Importantly, this level of shear stress is significantly higher than that in other vascular beds in the body.^[^ ^25^, ^26^ ^]^ Shear stress may aid in the vasodilation of LMCs to promote perfusion to the penumbra;^[^ ^27^ ^]^ however, hypertensive animals exhibit both poorer baseline LMC blood flow and impaired flow‐mediated vasodilation,^[^ ^24^, ^28^ ^]^ ultimately leading to poor penumbral perfusion. These observations raise the possibility that if one could selectively target a vasodilator to LMCs that experience high shear stress, this could provide a way to enhance LMC flow and thereby reduce stroke morbidity and mortality.

To approach this challenge, we leveraged a previously described drug delivery platform composed of spray‐dried, drug‐loadable, polymeric nanoparticles (≈180 nm diameter) that are formed into platelet sized (≈3 µm diameter) nanoparticle aggregates (NPAs). Importantly, these NPAs selectively release the nanoparticles and their contents where they experience pathological fluid shear stress (>100 dyne cm^−2^) when injected systemically into the circulation.^[^ ^29^ ^]^ When these shear‐activated NPAs were loaded with tissue plasminogen activator (tPA) and injected intravenously, they dissolved pulmonary emboli using one hundredth the dose of soluble tPA required to accomplish the same response in a mouse model.^[^ ^29^ ^]^ These tPA‐loaded NPAs also enhanced the removal of blood clots in large brain arteries through enzymatic dissolution.^[^ ^30^ ^]^ In the present study, we loaded these shear‐activated NPAs with nitroglycerin (NG‐NPAs) to explore whether we could selectively target this potent vasodilator to the smaller LMCs surrounding the penumbra in the context of ischaemic stroke. Our results show that NG‐NPAs can significantly enhance LMC blood flow, improve perfusion of the penumbra during stroke, decrease infarct volume, and reduce neurological deficit without causing systemic side effects that limit the use of free nitroglycerin for this life‐threatening condition.

## 2 Results

### 2.1 Increased WSS in LMCs in Rats and Humans

**Figure 1:**
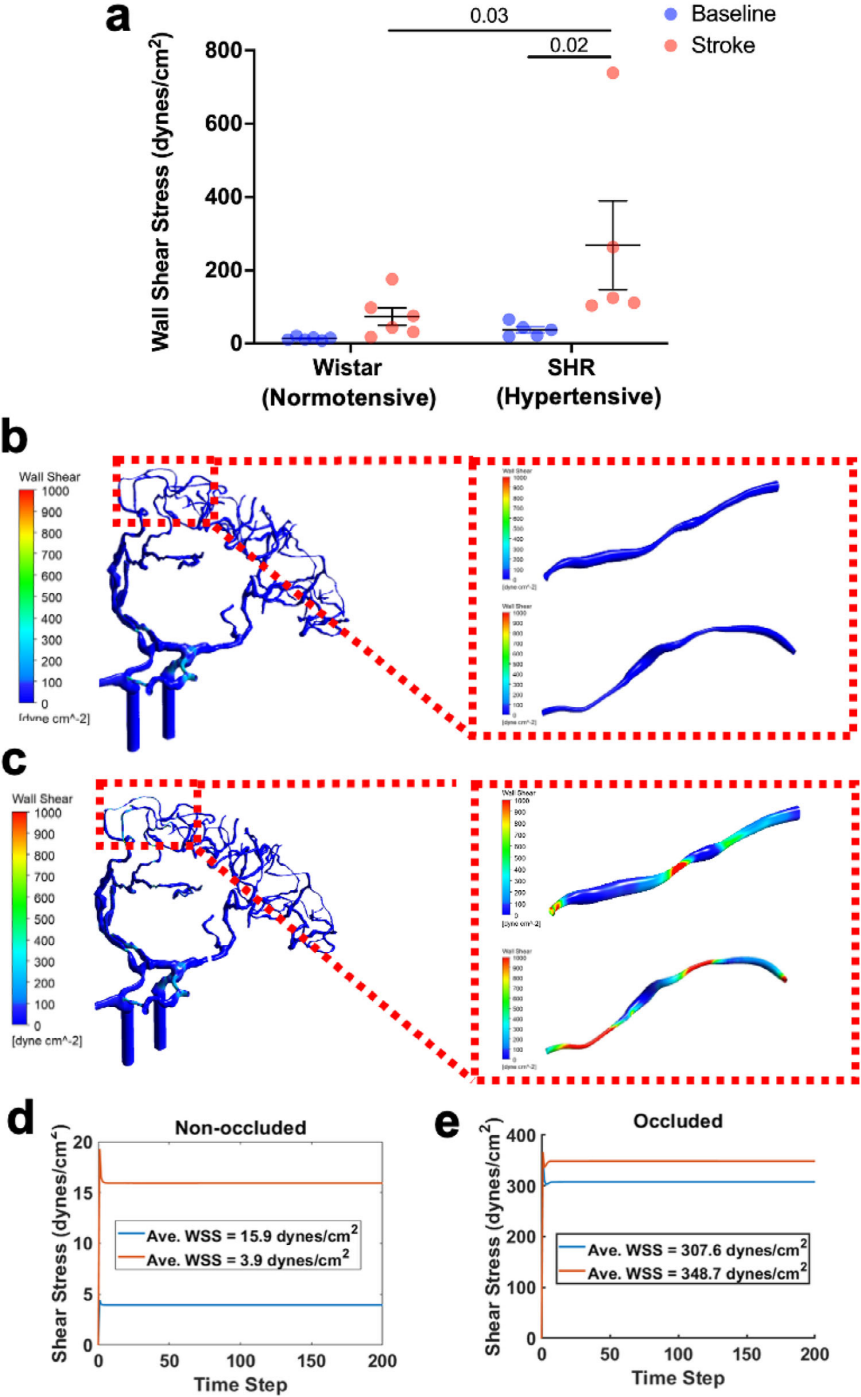
Wall shear stress (WSS) is significantly increased in LMC during stroke. a) Average WSS in collateral vessels in Wistar rats and spontaneous hypertensive rats. We conducted a repeated measure 2‐Way ANOVA to assess the effect of Strain (Wistar vs SHR) and Stroke on WSS. F (1,9) = 3.645, *p* = 0.09 for strain, F(1,9) = 6.852, *p* = 0.03 for stroke, F (1,9) = 2.361, *p* = 0.16. Uncorected Fisher's LSD test was used to compare the difference between baseline and stroke within and between Wistars and SHRs (*p* values on graph in italics, *p* < 0.05 between baseline and stroke in SHRs and stroke between Wistars and SHRs). b) Computational Fluid Dynamics (CFD) modelling of WSS in human cerebral vessels without occlusion of the proximal MCA (unoccluded model), with more detailed WSS analysis in individual collateral vessels (inset). c) Similar CFD modelling with occlusion of the proximal MCA (occluded stroke model). d) Quantified average WSS over time in individual collateral vessels (the WSS in each collateral is represented by a red and blue line) from the unoccluded model. e) Quantified average WSS over time in individual collateral vessels (the WSS in each collateral is represented by a red and blue line) from the occluded model. The WSS is color coded to represent dyne cm^−2^ as per the scale. Presented values represent mean ± SEM.

To determine the translational potential of our shear targeted drug delivery concept, we quantified fluid WSS levels in LMCs of both healthy normotensive Wistar rats and spontaneously hypertensive rats (SHRs). We included hypertensive rats because high blood pressure is one of the most common co‐morbidities and the leading modifiable risk factor for stroke, and it is associated with poor LMC blood flow.^[^ ^31^, ^32^ ^]^ We found that before the stroke, LMC blood flow was bi‐directional and very slow. However, upon induction of stroke by blocking the proximal segment of the MCA, flow became unidirectional. The blood flowed from the anterior cerebral artery (ACA) toward the MCA and the velocity increased dramatically while vessel diameter only increased slightly. When we quantified WSS in LMC following experimental stroke, we detected a 5‐ to 6‐fold increases in both normotensive Wistar rats (Baseline: 13.69 ± 1.89 dynes cm^−2^ vs Post‐Stroke: 73.73 ± 23.67 dynes cm^−2^, p = 0.4 and SHRs (Baseline: 43.8 ± 11.6 dynes cm^−2^ vs Post‐Stroke: 252.2 ± 100.4 dynes cm^−2^, p = 0.02. There were no significant differences between baseline LMC WSS between Wistar and SHR (p = 0.76). SHRs had significantly higher LMC WSS post‐stroke, compared to Wistars (p = 0.03) (**Figure** **1**a).

### 2.2 Computational Fluid Dynamics Analysis of Blood Flow in Human LMCs

To explore the potential clinical relevance of this observation, we carried out computational fluid dynamics (CFD) simulations of the LMCs following a simulated ischaemic stroke in a human. CTA images of a patient who had an ischaemic stroke with proximal MCA occlusion were segmented to produce a 3D model of the ACA, MCA, and LMCs that link them before (Figure 1b) and after (Figure 1c occlusion. When we simulated WSS in two LMCs (the WSS in each collateral is represented by a red and blue line in Figure 1d,e) under non‐occluded (Figure 1d) and occluded (Figure 1e) (ischaemic stroke following MCA occlusion) conditions, we found that there were 30‐ to 90‐fold increases in shear stress following occlusion of the LMCs.

### 2.3 Shear‐Activated Delivery of Nitroglycerin Selectively Increases LMC Perfusion

**Figure 2:**
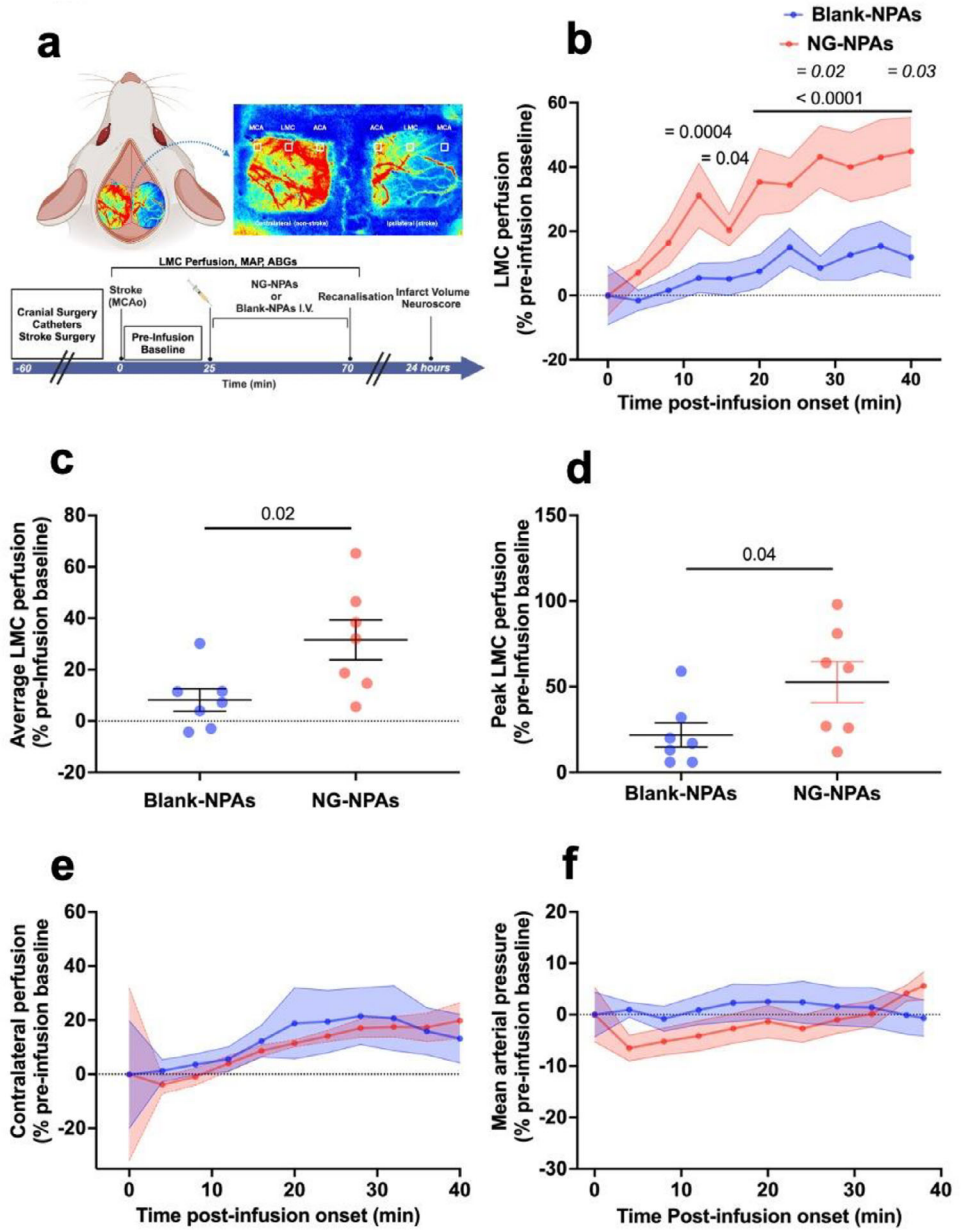
NG‐NPA infusion significantly increases LMC perfusion without causing changes in contralateral collateral perfusion or a dro*p* in mean arterial pressure in spontaneously hypertensive rats. a) Diagram demonstrating perfusion regions analysed using laser speckle contrast imaging during experimental stroke and experimental timeline. b) LMC perfusion in the stroke region in NG‐NPA (red) treated versus Blank‐NPA (blue) treated SHRs, calculated as a % change from pre‐drug infusion baseline. We conducted a repeated measure 2‐Way ANOVA to assess the effect of NG‐NPA versus Blank‐NPA treatment over time. F (1, 12) = 6.936, *p* = 0.02 for treatment, F (10, 120) = 8.035, *p* < 0.0001 for time, F (10, 120) = 2.1, *p* = 0.03 for interaction. Sidak's post‐test was used to compare NG‐NPA versus Blank‐NPAs at different time‐points (*p* values on graph in italics, *p* < 0.05 at 30 and 40 min). Dunnett's post‐test was used to compare each time‐point back to pre‐infusion baseline in NG‐NPA and Blank NPA groups (*p* values on graph in normal text, NG‐NPA: *p* < 0.05 at 16 min; *p* < 0.001 at 12 min; *p* < 0.0001 between 20 and 40 min). c) Average perfusion within the LMC region over time, for each animal. We used an un‐paired *t*‐test to assess differences between NG‐NPAs (red dots) versus Blank‐NPAs (blue dots). T (12) = 2.63, *p* = 0.02 versus Blank‐NPAs. d) Peak perfusion within the LMC region for each animal. We used an un‐paired *t*‐test to differences between NG‐NPAs (red dots) versus Blank‐NPAs (blue dots). T (12) = 2.212, *p* = 0.04 versus Blank‐NPAs. e) LMC perfusion in the contralateral (control) hemisphere in NG‐NPA (red) treated versus Blank‐NPA (blue) treated SHRs. We conducted a repeated measure 2‐Way ANOVA to assess the effect of NG‐NPA versus Blank‐NPA treatment over time. F (1,12) = 0.156, *p* = 0.69 for treatment, F (10, 120) = 1.35, *p* = 0.21 for time, F (10, 120) = 0.08, *p* > 0.99 for interaction. f) Mean Arterial Pressure in NG‐NPA (red) treated versus Blank‐NPA (blue) treated. We conducted a repeated measure 2‐Way ANOVA to assess the effect of NG‐NPA versus Blank‐NPA treatment over time. F (1,12) = 0.49, *p* = 0.5 for treatment, F (10, 120) = 1.02, *p* = 0.42 for time, F (10, 120) = 1.49, *p* = 0.15 for interaction. Values represent mean ± SEM.

As hypertensive patients are the most difficult to treat, and we observed the greatest level of shear stress in SHRs, all further experiments were conducted using the hypertensive animals. MCA occlusion was induced for 70 min in male SHRs, and laser speckle contrast imaging was used to measure tissue perfusion in the LMCs (**Figure** **2**a). Animals were randomised to receive intravenous infusion of NPAs without drug (Blank‐NPAs) or NG‐NPAs (4 µg NG kg^−1^ min^−1^) commencing 25 min after the MCA was blocked, and infarct volume was measured at 24 h (Figure 2a). These studies revealed that infusion of NG‐NPAs increased LMC perfusion by almost 45% between 12 and 40 min compared to pre‐infusion baseline, while there was no significant change in LMC perfusion compared to pre‐infusion baseline when the same dose of Blank‐NPAs was administered (Figure 2b). The mean perfusion within the LMC region was calculated for each animal over the entire infusion period and found to be significantly higher in the NG‐NPAs compared the Blank‐NPAs group (Figure 2c).

We also identified the highest‐level perfusion in the LMC region at any time‐point throughout infusion in each animal (peak). This was also found to be significantly higher in NG‐NPAs versus Blank‐NPAs (Figure 2d).

In contrast, systemic administration of the shear targeted NG‐NPAs did not significantly alter perfusion in the LMC region in the contralateral (non‐stroke/control) hemisphere compared to pre‐infusion baseline or relative to Blank‐NPAs (Figure 2e). Importantly, infusion of the NG‐NPAs also did not reduce systemic blood pressure relative to pre‐infusion baseline or Blank‐NPAs (Figure 2f). All other physiological variables were within physiological range and were not significantly different between treatment groups (**Table** **1**). Thus, the shear‐activated NG‐NPAs appeared to selectively produce vasodilation and enhanced perfusion precisely where our rat experiments and human models predicted fluid shear stress to be preferentially elevated.

**Table 1:** Physiological parameters measured pre‐and‐during NG‐NPA versus Blank‐NPA infusion from Study 1. Mean ± SEM of MAP, mean arterial pressure; RR, respiratory rate; HR, heart rate; SPO_2,_ oxygen saturation; paO_2,_ arterial partial pressure of oxygen; paCO_2_, arterial partial pressure of carbon dioxide.

	NG‐NPAs		Blank‐NPAs	
	Pre‐infusion	During infusion	Pre‐infusion	During infusion
MAP (mmHg)	158.7 ± 10.56	155 ± 10.27	158.8 ± 8.18	171.7 ± 7.36
RR (BMP)	52.14 ± 2.77	53.10 ± 2.68	45.21 ± 2.09	44.88 ± 2.87
HR (BPM)	398.4 ± 12.28	407.3 ± 14.86	393.6 ± 11.91	417.3 ± 12.86
SPO2 (%)	95.89 ± 1.03	97.11 ± 1.06	95.74 ± 0.72	96.5 ± 0.54
paO2 (mmHg)	97.25 ± 8.11	99.50 ± 5.87	114.5 ± 6.84	112.5 ± 9.18
paCO2 (mmHg)	43.40 ± 0.26	41.45 ± 0.71	41.03 ± 1.53	39.65 ± 1.14
pH	7.387 ± 0.0056	7.399 ± 0.007	7.414 ± 0.01	7.39 ± 0.009

### 2.4 Shear‐Targeted Delivery of Nitroglycerin Improves Stroke Outcomes

**Figure 3:**
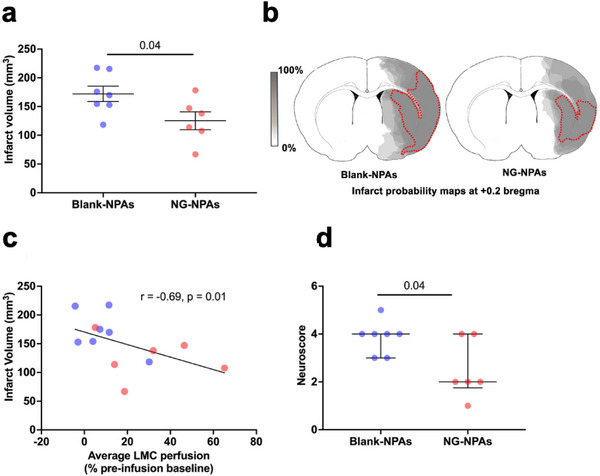
NG‐NPAs significantly reduced infarct volume and improved neurological function 24 h post stroke in SHRs. a) Infarct volume was assessed histologically at 24 h after stroke onset. We used an un‐paired *t*‐test to differences between NG‐NPAs (red dots) versus Blank‐NPAs (blue dots). T (11) = 2.29, *p* = 0.04 versus Blank‐NPAs. b) Infarct probability maps were generated from infarct areas taken from the brain region in the central region supplied by the middle cerebral artery (+0.2 bregma brain slice). Darker grey regions represent areas with a higher probability of infarction. c) Spearman's correlation between infarct volume and average LMC perfusion. d) Neurological score assessing forelimb flexion, lateral push, and torso twisting (0 = normal, 6 = maximal deficit). We used a Mann‐Whitney U test to to assess differences between NG‐NPAs (red dots) versus Blank‐NPAs (blue dots). U = 9, *p* = 0.03. Values represent mean ± SEM.

At 24 h after stroke onset, animals underwent assessment of neurological deficits, and they were then euthanised, perfusion fixed, and brains were collected for coronal sectioning and histological evaluation of infarct size using haematoxylin and eosin staining. Quantification of infarct size revealed that NG‐NPAs significantly reduced final infarct volume by over 35% compared to Blank‐NPAs (**Figure** **3**a). Infarct probability maps also indicated that a reduction in the frequency of infarcts in the cortex located downstream of the LMC (i.e., in the penumbral region) is likely responsible for the reduction in overall infarct volume in the NG‐NPA group (Figure 3b). There was a very large and significant inverse correlation between the average level of LMC perfusion and infarct volume (spearman's r = ‐0.69, p = 0.01; Figure 3c). The neurological deficit scores also were significantly lower in animals receiving NG‐NPAs (median score = 2; range 1–4) versus Blank‐NPAs (median = 4; range 3–5) (Figure 3d).

### 2.5 Shear‐Targeted Delivery Did Not Cause Detectable Adverse Effects

**Figure 4:**
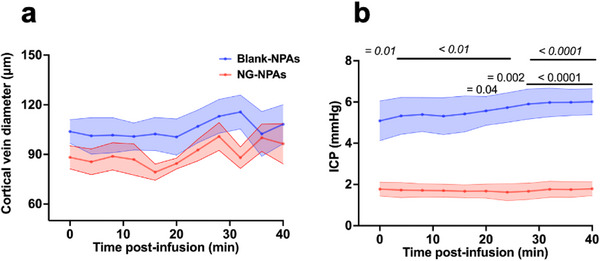
NG‐NPAs did not cause changes in cortical vein diameter or ICP in SHRs. a) Cerebral vein diameter following NG‐NPA (red dots) or Blank‐NPA (blue dots) administration. We conducted a Mixed‐Effects Model to assess the effect of NG‐NPA versus Blank‐NPA treatment over time. *p* = 0.2 for treatment, *p* = 0.002 for time, *p* = 0.4 for interaction. b) ICP following NG‐NPA (red dots) or Blank‐NPA (blue dots) administration. We conducted a repeated measure 2‐Way ANOVA to assess the effect of NG‐NPA versus Blank‐NPA over time. F (1, 5) = 16.38, *p* = 0.001 for treatment, F (10, 50) = 3.7, *p* < 0.0009 for time, F (10, 50) = 3.428, *p* = 0.002 for interaction. Sidak's post‐test was used to compare NG‐NPA versus Blank‐NPAs at different time‐points (*p* values on graph in italics, *p* = 0.01 at 0 min; *p* < 0.01 between 4 and 24 min, and *p* < 0.0001 between 28 and 40 min). Dunnett's post‐test was used to compare each time‐point back to pre‐infusion baseline in NG‐NPA and Blank NPA groups (*p* values on graph in normal text, Blank‐NPAs: *p* = 0.04 at 20‐min; *p* = 0.002 at 24 min, and *p* < 0.0001 between 28 and 40 min). Values represent mean ± SEM.

NG administration as a free drug has previously been shown to have adverse effects due to dilation of cerebral veins, which increases intracranial pressure.^[^ ^15^ ^]^ Importantly, when we infused the NG‐NPAs, we did not detect any change in cortical vein diameter compared to pre‐infusion baseline or relative to administration of Blank‐NPAs (**Figure** **4**a). The NG‐NPA treatment also did not change ICP compared to pre‐infusion baseline (Figure 4b), and it was significantly lower in the NG‐NPA group compared to Blank‐NPAs at all time‐points, including pre‐infusion baseline (Figure 4b).

### 2.6 Administration of Free NG is Ineffective

**Figure 5:**
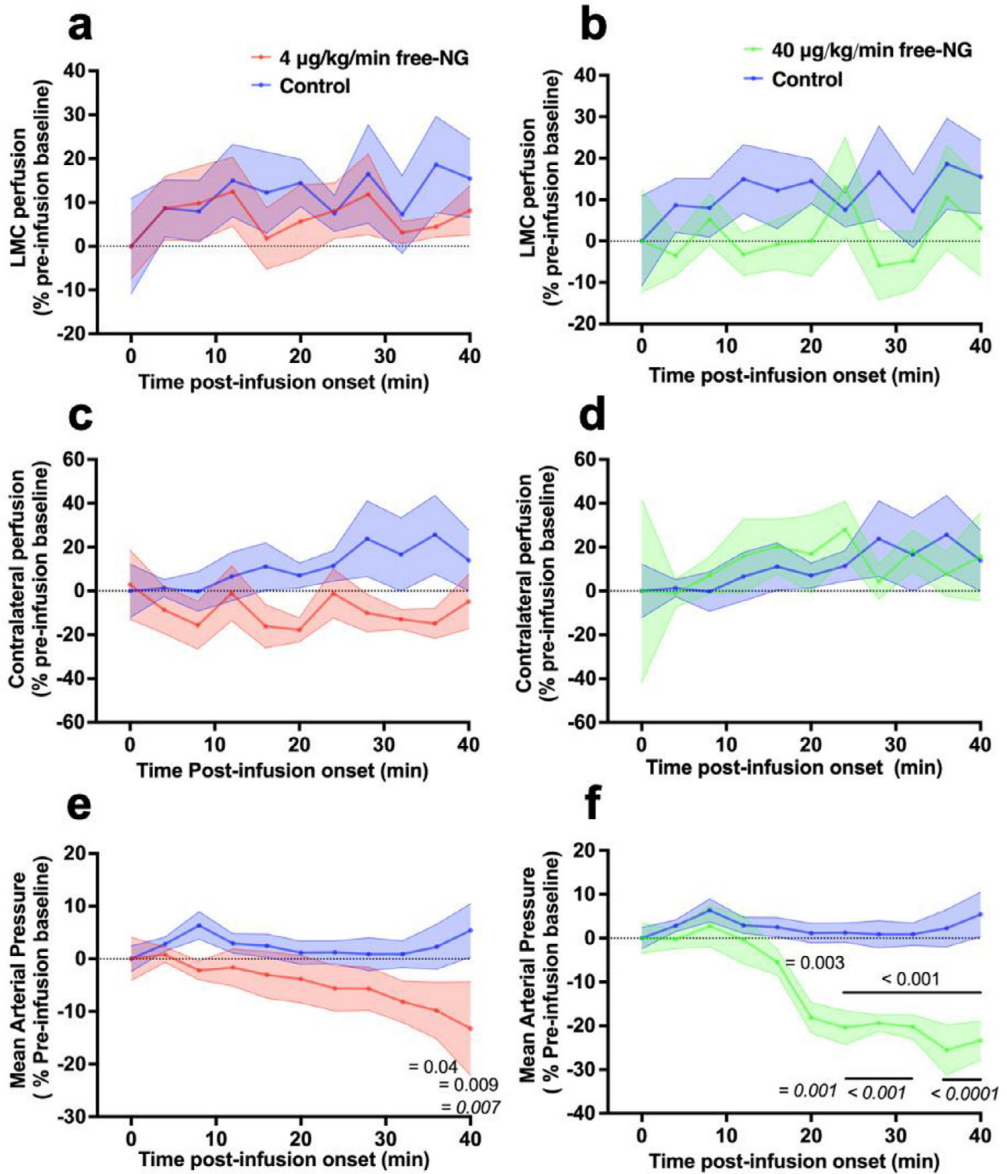
Administration of free‐NG did not improve LMC perfusion but caused a dro*p* in mean arterial pressure. a) The effect of 4 µg kg min^−1^ free‐NG on ipsilateral LMC perfusion. We conducted a Mixed‐Effects Model to assess the effect of 4 µg kg min^−1^ free‐NG versus saline control over time. *p* = 0.69 for treatment, *p* = 0.52 for time, *p* = 0.98 for interaction. b) The effect of a 10 times higher dose (40 µg kg min^−1^) of free‐NG on ipsilateral LMC perfusion. We conducted a Mixed‐Effects Model to assess the effect of 40 µg kg min^−1^ free‐NG versus saline control over time. *p* = 0.22 for treatment, *p* = 0.8 for time, *p* = 0.85 for interaction. c) The effect of 4 µg kg min^−1^ free‐NG on contralateral perfusion. We conducted a Mixed‐Effects Model to assess the effect of 4 µg kg min^−1^ free‐NG versus saline control over time. *p* = 0.09 for treatment, *p* = 0.96 for time, *p* = 0.66 for interaction. d) The effect of high dose (40 µg kg min^−1^) free‐NG on contralateral perfusion. We conducted a Mixed‐Effects Model to assess the effect of 40 µg kg min^−1^ free‐NG versus saline control over time. *p* = 0.82 for treatment, *p* = 0.83 for time, *p* = 0.96 for interaction. e) The effect of 4 µg kg min^−1^ free‐NG on mean arterial pressure. We conducted a Mixed‐Effects Model to assess the effect of 4 µg kg min^−1^ free‐NG versus saline control over time. *p* = 0.07 for treatment, *p* = 0.89 for time, *p* = 0.04 for interaction. Sidak's post‐test was used to compare 4 µg kg min^−1^ free‐NG versus saline control at different time‐points (*p* values on graph in italics, *p* = 0.007 at 40 min). Dunnett's post‐test was used to compare each time‐point back to pre‐infusion baseline in 4 µg kg min^−1^ free‐NG and saline control groups (*p* values on graph in normal text, 4 µg kg min free‐NG: *p* = 0.04 at 36 min; *p* = 0.009 at 40 min). f) The effect of 40 µg kg min^−1^ free‐NG on mean arterial pressure. We conducted a repeated measure 2‐Way ANOVA to assess the effect of 40 µg kg min^−1^ free‐NG versus saline control over time. *p* = 0.002 for treatment, *p* < 0.0001 for time, *p* < 0.0001 for interaction. Sidak's post‐test was used to compare 40 µg kg min^−1^ free‐NG versus saline control at different time‐points (*p* values on graph in italics, *p* = 0.001 at 20 min; *p* < 0.001 between 24 and 32 min; *p* < 0.0001 between 36 and 40 min). Dunnett's post‐test was used to compare each time‐point back to pre‐infusion baseline in 40 µg kg min^−1^ free‐NG and saline control groups (*p* values on graph in normal text, 40 µg kg min^−1^ free‐NG: *p* = 0.0003 at 20 min; *p* < 0.0001 between 24 and 40 min). Values represent mean ± SEM.

We next wanted to confirm that the LMC perfusion‐increasing effects of the shear‐targeted NG‐NPAs we observed could not be obtained using a similar dose of free NG (4 µg kg min^−1^). Infusion of the same dose of free NG did not significantly change LMC perfusion relative to pre‐infusion baseline or to saline controls in the region of the stroke (**Figure** **5**a) or in the LMCs within the contralateral (non‐stroke/control) hemisphere (Figure 5c). Even when we administered 10 times the dose of free NG (40 µg kg min^−1^), we could not detect a significant change in perfusion in LMCs in the stroke area (Figure 5b) or in the LMCs in the contralateral hemisphere (Figure 5d).

Most concerning was that infusion of an equivalent amount of free NG significantly lowered systemic blood pressure 40 min post‐infusion relative to pre‐infusion baseline (Figure 5e), as observed in patients who received systemic administration of NG for treatment of stroke.^[^ ^20^ ^]^ As expected, this was further exacerbated by administration of the 10‐fold higher dose of free NG (Figure 5f). Thus, this animal model replicates clinical observations seen in stroke patients treated with free NG, and it shows that free NG cannot provide therapeutic effects for ischaemic stroke like those produced when NG is targeted to regions of high shear stress using the NPA delivery system.

## 3 Conclusion

Modulation of LMC perfusion has been recognized as a potentially exciting therapeutic approach to ischaemic stroke in human patients; however, producing this response without dangerous side effects has proven elusive. A key reason has been the inability to selectively enhance perfusion within relevant LMCs while avoiding systemic vasodilation and resultant hypotension. In this study, we showed that fluid WSS in LMC vessels in the region downstream of the occlusion is significantly higher during ischaemic stroke in both normotensive and hypertensive animals, as well as in humans, based on computational modelling.

In normal human circulation, WSS is typically below 100 dyne cm^−^^2^.^[^ ^33^, ^34^ ^]^ Nevertheless, it is important to note that shear‐induced disaggregation is not an all‐or‐nothing phenomenon occurring at a fixed threshold (e.g., 100 dyne cm^−^^2^), but rather a gradual process that begins ≈100 dyne cm^−^^2^ and intensifies with increasing shear stress.^[^ ^29^, ^35^ ^]^ This behaviour aligns with the hemodynamic environment of stenotic arteries, where localized wall shear stress can exceed 1000 dyne cm^−^^2^.^[^ ^34^ ^]^ In this study, we demonstrate that similarly elevated wall shear stress levels can occur in LMC vessels during stroke, both in humans and in the animal models analysed. Since shear‐induced release is dose‐dependent, significantly greater release occurs under higher WSS conditions. Additionally, shear stress in flowing blood exhibits a radial gradient—peaking at the vessel wall and approaching zero at the center. As a result, wall shear stress defines a broad perivascular region where particles are exposed to sufficiently high shear levels, thereby amplifying the release response. Moreover, in this work, we show that this hemodynamic peculiarity can be leveraged using a shear‐activated nanoparticle drug delivery system to selectively deliver a potent FDA‐approved vasodilator (NG) to these sites and thereby, enhance penumbral perfusion, while avoiding deleterious side effects.

This mechanotherapeutic approach is supported by the finding that fluid WSS increases abruptly immediately after the MCA is experimentally occluded in normotensive and hypertensive rats, as shown here, as well as in normotensive mice.^[^ ^23^ ^]^ However, fluid WSS measured in the LMCs of the hypertensive rats was significantly higher after stroke than that in normotensive animals, increasing to values well above the threshold of activation for NG‐NPAs to release their contents. This is highly relevant from a translational perspective, as the normotensive Wistar rats we used, have larger diameter LMC vessels and smaller infarcts than the SHRs.^[^ ^28^, ^36^ ^]^ Hypertension is also seen in the majority of stroke patients,^[^ ^37^ ^]^ and it is associated with poor collateral blood flow and worse outcomes in both patients^[^ ^38^ ^]^ and animals.^[^ ^39^ ^]^ Therefore, hypertensive patients would be more likely to respond to and have the most to gain from the NG‐NPA treatment, therefore this could be a choice patient population for future clinical studies.

As a control in this study, we measured perfusion through LMCs in the same animal on the contralateral side of the brain that did not experience stroke and found that infusion of the NG‐NPAs had no effect on their perfusion. Most importantly, the specific enhancement of perfusion through the stroke associated LMCs resulted in a significant reduction in infarct size, consistent with clinical observations suggesting that better collateral perfusion is associated with smaller infarct volumes.^[^ ^40^ ^]^ Our findings also demonstrated that targeted delivery of NG to the LMCs in stroke regions using the shear‐activated NPA delivery platform led to selective collateral vasodilation without producing systemic hypotension or increased intracranial pressure. In fact, ICP was lower in the NG‐NPA group versus blank at pre‐infusion baseline and all other time points. ICP values were within the expected physiological range (1‐7 mmHg) reported in our previous studies using our well‐established technique.^[^ ^41^, ^42^ ^]^ These results suggest that systemic arterial vasodilation and cerebral venodilation also did not occur.

Systemically administered NG has been previously proposed as a potential therapeutic agent in the treatment of acute ischaemic stroke, and it has been administered as a transdermal patch in seven randomised clinical trials.^[^ ^43^, ^44^, ^45^, ^46^, ^47^, ^48^, ^49^ ^]^ The recently published RIGHT‐2 trial (2019) showed that transdermal NG was effective in lowering systemic blood pressure but was neutral for functional outcome at 3 months post‐stroke. In fact, the point estimates indicated a trend for harm in patients with the final diagnosis of stroke or transient ischaemic attack.^[^ ^46^ ^]^ As perfusion imaging was not performed on trial patients, the reasons for these results are unclear. A possible explanation for this is that the drop in systemic blood pressure counteracted the benefits of cerebral vessel dilation caused by NG. This is supported by the findings from another study measuring changes in cerebral perfusion associated with transdermal NG treatment. Of the 18 patients treated with transdermal NG within 5 days of symptoms onset, blood pressure was reduced, and cerebral blood flow remained unaffected.^[^ ^43^ ^]^ A subsequent perfusion study using the same treatment protocol as the RIGHT‐2 trial showed no improvement in penumbral perfusion on imaging, suggesting that the drug might not have made it to the brain or, if it did, the therapeutic concentration in the brain was not reached, resulting in no change in cerebral perfusion.^[^ ^50^ ^]^ Thus, our finding that intravenous infusion of free NG using an equivalent dose to what was in the NPAs did not enhance collateral perfusion, but caused systemic vasodilation and systemic hypotension, is consistent with known effects observed in human patients.^[^ ^48^, ^51^ ^]^ Furthermore, administration of a 10 times higher dose of free NG did not affect tissue perfusion in this experimental model, but significantly lowered systemic blood pressure, suggesting that any effect of dilation of collaterals on blood flow was counteracted by the reduction in blood pressure. Because of these dose‐limiting hypotensive effects, we could not find a dose of free NG that produced the equivalent collateral enhancing effect to NG‐NPAs.

In contrast, our results suggest that by selectively targeting short‐acting vasodilators to LMCs using the shear‐activated NPA delivery platform, only a low dose of active NG drug is needed to increase penumbral perfusion, reduce infarct size, and decrease neurological deficit, while avoiding systemic complications. It is important to note that the total dose of NG (0.16 mg kg^−1^) we loaded in the NG‐NPAs, was much lower than that used in other preclinical studies that were looking at alternative ways to use free NG for the treatment of stroke. For example, in one study that was designed to target NG to the cerebral vasculature and limit systemic side effects, by direct intraarterial administration through the internal carotid artery following reperfusion in a mouse stroke model, they found that only the two highest doses (2 and 4 mg kg^−1^) that were more than 10 times that dose loaded in our NG‐NPAs were found to reduce infarct volume and improved functional outcome; however, even then they were not able to demonstrate improvement in CBF.^[^ ^52^ ^]^ Reduced infarct volume has also been shown with NG administered at a very high dose (10 mg kg^−1^, i.p.) 20 min pre‐stroke in rats, but the same study failed to show benefits with post‐stroke administration.^[^ ^53^ ^]^ Unfortunately, while overall the results were positive, the treatment window, route of administration, and very high doses of drug required in both studies are not relevant for use in clinical practice. A more recent study using treatment with a NG patch loaded with doses equivalent to human trials (0.06 mg kg^−1^) failed to show significant improvement of cerebral blood flow, infarct size or functional outcome in a mouse model of ischaemic stroke, in line with clinical data.^[^ ^54^ ^]^ In that study, increasing the NG doses to up to 48 mg kg^−1^ (700 times the clinical dose) did not produce any additional benefit on cerebral blood flow, infarct size or functional outcome. Furthermore, a recent systematic review and meta‐analysis of these experimental studies of NG in stroke, revealed an overall neutral effect of systemic NG on infarct volume.^[^ ^55^ ^]^ Thus, our results with NG‐NPAs are both highly novel and clinically relevant.

Although our results demonstrate the ability of NG‐NPAs to selectively enhance collateral‐mediated penumbral perfusion in animals with ischaemic stroke and hypertension, we acknowledge that further work will be needed to confirm benefits in a more easily achievable clinical time‐window. NG‐NPAs were infused at 25 min post vascular occlusion because of constraints due to very rapid infarct expansion seen in SHRs. Hence, to conduct studies in the most humane manner, we did not wish to delay drug infusion to the point where it resulted in very large infarcts. However, it should be noted that this is a therapy with strong potential to be delivered in‐ambulance, in which case a 25 min time interval may be achievable in some patients, and a median treatment time of 45 min from stroke onset was reported in the FAST‐MAG trial in 2015.^[^ ^56^ ^]^ More recently, the RIGHT II and MR ASAP trials, conducted in Europe achieved median administration times of 73 and 63 min with transdermal NG, respectively.^[^ ^44^, ^46^ ^]^

Furthermore, a comprehensive toxicity analysis of our NG‐NPA formulation will need to be conducted prior to clinical translation. Although assessment of the systemic toxicity of NG‐NPAs was beyond the aims of the current study we do not anticipate any toxicity related issues for either the NPAs or NG accumulating in the liver or spleen as the doses of nitroglycerin used (total NG administered = 4 µg kg min^−1^ and 160 µg kg total^−1^) are well below the reported NOAEL doses of oral NG for hepatic toxicity (24 µg kg min^−1^, 35 000 µg kg day^−1^ for 12 months, which is exclusively delivered and metabolised in the liver).^[^ ^57^ ^]^ Further, the doses of NPAs used (13 mg kg^−1^) is far below doses that have previously been shown to be safe in mice (160–2400 mg kg^−1^ for between 1–10 days).^[^ ^58^ ^]^ We acknowledge that the novel formulation of our NPAs combined with NG may pose unknown toxicity issues and thus will form the basis of future work investigating the safety of our approach.

In conclusion, our results demonstrate that treatment with NG‐NPAs significantly increased LMC‐mediated penumbral perfusion, decreased infarct volume, and reduced neurological deficits 24 h after induction of experimental stroke in hypertensive rats with poor baseline collateral status. Administration of free NG at the dose equivalent to that in NG‐NPAs did not show benefit in enhancing collateral blood flow and instead caused significant hypotension, as is also observed clinically in stroke patients who were administered with free NG. We suggest that selective targeting of the collateral vessels using NG‐NPAs based on their unique shear stress profile during stroke may realise the long‐recognised potential of using widely available vasodilators to improve stroke outcomes. This would be accomplished via shear targeted delivery to LMCs thereby enhancing collateral flow and penumbral perfusion, while avoiding counteracting effects on blood flow due to systemic vasodilation and hypotension. Ultimately, if these effects are confirmed in patients, there would be potential benefits to “buy time” prior to administration of reperfusion therapies, in addition to providing hope for those ineligible or unsuitable for such therapies.

## 4 Experimental Section

### Animal Studies

Spontaneously hypertensive rats (SHR male, 12–14 weeks old n = 56), and normotensive Wistars (male, 12–14 weeks, n = 6) were used in this study. Experiments conformed to the Animal (Scientific Procedures) Act 1986 (United Kingdom), the National Institutes of Health guidelines for care and use of laboratory animals, and were approved by the University of Oxford Animal Ethics Committee, the Home Office (United Kingdom). Experiments were approved by the Animal Care and Ethics Committee of the University of Newcastle, Australia (Protocol #A‐2020‐003 and Protocol #A‐2011‐131, respectively), in accordance with the requirements of the Australian Code of Practice for the Care and Use of Animals for Scientific Purposes. The studies were conducted, and the article was prepared in accordance with the STAIR recommendations^[^ ^59^ ^]^ and reported in accordance with the ARRIVEguidelines.^[^ ^60^ ^]^ Detailed animal and experimental descriptions were available in the Supporting Information.

### Quantification of Fluid Wall Shear Stress in LMCs

Fluid wall shear stress (WSS) was measured in the LMCs of normotensive Wistar rats and SHRs by intravenously infusing fluorescent microspheres (1 µm in diameter, 0.2% w v^−1^; Molecular Probes) at the rate of 4 mL h^−1^ and measuring their movement through the LMC between the anterior cerebral artery (ACA) and MCA through a cranial window using an Olympus BX60 microscope at 10X magnification. LMC flow was recorded using a fluorescent microscope‐mounted high‐speed camera (Ace U acA720‐520um, Basler, Germany or Genie HM640, Teledyne Dalsa, Canada) at 300 frames s^−1^.^[^ ^22^, ^61^ ^]^ This allowed us to measure LMC blood flow velocity and LMC vessel diameter. Collateral velocity and diameter data have previously been reported for the Wistar and SHR cohort of animals; however, WSS was not previously analysed. We used these parameters to quantify total WSS in these collateral vessels using the Equation: τ = γ × η, where τ was shear stress, γ was shear rate, and η was viscosity. Shear rate was calculated as 8 × (blood flow velocity) / (vessel diameter). We used published figures for blood viscosity (3 cP).^[^ ^23^ ^]^

For this calculation and the computational fluid dynamics analysis, blood was modeled as a Newtonian fluid because shear rates in arteries typically exceed 100 s^−1^, minimizing shear‐thinning effects and yielding WSS predictions similar to non‐Newtonian models, thereby supporting the use of a Newtonian viscosity in the simulations to simplify the model and improve computational efficiency.^[^ ^62^, ^63^ ^]^

### Computational Fluid Dynamics Analysis

A 3D cerebrovascular model was generated from computed tomography angiogram (CTA) images of a patient who had suffered an ischaemic stroke, selected from the International Stroke Perfusion Imaging Registry (INSPIRE). Patients were prospectively enrolled as part of the INSPIRE, approved by the Hunter New England Local Health District Human Research Ethics Committee in accordance with Australian National Health and Medical Research Council guidelines (Reference No.: 11/08/17/4.01). For this study, an opt‐out consent was used.

These CTA images were segmented using a modelling and segmentation software 3D Slicer (http://www.slicer.org), according to the method adapted from Bateman et al.^[^ ^64^ ^]^ To reduce computational time, the cerebrovascular system consists of a complete arterial system of one hemisphere and a removed arterial system on the contralateral side (Figure S1A, Supporting Information). The length of the left and right internal carotid arteries (ICAs) of the model, which were the inlets to the system, was extended to allow the flow at the inlets to be fully developed before entering the cerebrovascular system. To implement an occlusion, a section of the proximal MCA was removed (Figure S1B, Supporting Information). A tetrahedron mesh was used to model the cerebral vasculature due to the complexity of the geometry. Mesh sensitivity analysis was conducted to ensure the convergence of solutions.

Flow conditions were applied to the inlet and outlet vessels according to flow rate values found in Padmos et al.^[^ ^65^ ^]^ For inlet velocity, the average flow rate passing through the ICA was ≈260 mL min^−1^. With a measured ICA diameter of 5.156 mm, this amounts to an average inlet velocity of 0.2075 m s^−1^, which was applied to the two inlets of ICAs (indicated by blue arrows in Figure S1A, Supporting Information). Outlets of the model were grouped into large vessel outlets and collateral outlets. The flow rates at the outlet of large vessels were obtained (Table S1, Supporting Information) and were setup using the target mass flow rate function within ANSYS Fluent (ANSYS, Inc, Canonsburg USA). A gauge pressure of 40 mmHg was set at the outlets of the remaining smaller vessels for the occluded model. For the non‐occluded model, the outlet pressure at the vessels was set to 0 mmHg. For the wall boundary condition, a rigid arterial wall with a no‐slip condition was applied. The shear stress transport fluid model was applied to simulate the complex cerebrovascular model with large variations in geometry.

Two LMC vessels were extracted from the full cerebrovascular model using CREO (PTC, Boston USA). The two collateral vessels were meshed with 429425 and 623531 elements respectively to analyse their WSS with higher details and accuracy. The average inlet velocity to each collateral was obtained from the simulated results of the full cerebrovascular models. The boundary conditions and average WSS for each collateral vessel can be found in Table S2 (Supporting Information).

### Nitroglycerin‐Loaded PLGA Nanoparticle (NG‐NPA) Fabrication and Characterization

Nitroglycerin‐ loaded PLGA (poly(lactic‐co‐glycolic acid)) nanoparticle aggregates (NG‐NPAs) were synthesized using a single emulsion solvent evaporation method. The aqueous phase was prepared by dissolving 1% (w v^−1^) poly(vinyl alcohol) (PVA; Sigma–Aldrich, Cat# 360 627) in Milli‐Q water, followed by sterile filtration through a 0.2 µm filter. The organic phase consisted of GMP‐grade PLGA (50:50 lactide:glycolide; Durect Lactel, Cat# B6013, MW 7–17 kD) dissolved in dichloromethane (DCM; Sigma–Aldrich, Cat# 32 222, Inherent Viscosity 0.15 – 0.25 dL gr^−1^) at a concentration of 50 mg mL^−1^. For standard preparations, 0.5 g of PLGA was dissolved in 10 mL of DCM in a sealed 20 mL scintillation vial under magnetic stirring. Nitroglycerin (NG) (5 mg mL^−1^; American Regent, Nitroglycerin Injection, USP) was added to the PLGA/DCM solution (10 mL), and the mixture was transferred into 50 mL of the prepared PVA solution in an ice‐cooled beaker. The emulsion was sonicated for 1.5 min at 40% amplitude using a probe sonicator (QSonica Q700, probe model CL‐334) inside a 4 °C cold room. One milliliter of the resulting emulsion was reserved for immediate nanoparticle size characterization via dynamic light scattering (DLS). The remaining emulsion was dialyzed overnight against Milli‐Q water using 100 kDa MWCO cellulose ester dialysis tubing (Spectrum Labs). After dialysis, samples were collected in 50 mL conical tubes. Aliquots (2 mL) were taken for quantifying NG loading via liquid chromatography–mass spectrometry (LC‐MS) and for particle size analysis by DLS. Nanoparticle yield was determined by drying triplicate 0.5 mL aliquots at 120 °C and averaging the dry weights. Finally, the nanoparticle suspension was adjusted to a concentration of 5 mg mL^−1^ using Milli‐Q water. The nanoparticles used to make NG‐NPAs had an average size of 189 ± 8.7 nm with a polydispersity index (PDI) of 0.1, as measured by DLS across 10 independent batches. Additionally, the NG loading was 1.83 ± 0.25% across the same 10 batches. The nanoparticles used to make Blank‐NPAs had an average size of 187 ± 4 nm (Table S3, Supporting Information).

### Fabrication of NG‐NPAs via Spray Drying and Their Characterization

NG‐NPAs were prepared using a Buchi B‐290 spray dryer. A 5 mg mL^−1^ L‐leucine solution was prepared by dissolving 0.5 –1 g of L‐leucine (Spectrum Chemicals & Laboratory Products, CA) in 100–200 mL of Milli‐Q water with stirring for 1 h. Prior to spray drying, the NG‐NPAs (prepared as described above) were mixed with 200 proof ethanol at a 1:1.5 (v/v) ratio immediately before the drying process. The Buchi B‐290 spray dryer was sterilized via autoclaving, and the system was initially run with deionized water to stabilize operating conditions. Once stable conditions were achieved (inlet temperature: 110–125 °C, outlet temperature: 35–40 °C, feed rate: 4–6 mL min^−1^, aspirator pressure: >60 mbar), the aqueous feed was switched to the L‐leucine solution. Upon visible coating of the collection container with leucine powder, indicating internal coating of the system, the feed was transitioned to the ethanol‐mixed NG nanoparticle suspension. Following completion of the suspension feed, the system was flushed with deionized water. The resulting spray‐dried microparticles, were collected at the cyclone outlet, scraped into 20 mL glass vials using a sterile dry spatula, and stored in a desiccator at −20 °C until further use. The average size of the NG‐NPAs (measured by laser diffraction, Malvern) was 2.2 ± 0.5 µm across 10 independent batches. Drug loading was 1.1 ± 0.4%, as measured from the same 10 batches (Table S3, Supporting Information). Blank‐NPAs were also manufactured using the same process, applied to nanoparticles that were not loaded with any drug. The average size of the Blank‐NPAs was 2.7 ± 0.25 µm across 3 independent batches. (Table S3, Supporting Information).

### Fabrication of NG‐NPAs via Spray Drying and Their Characterization—In Vitro Drug Release from NG‐NPAs

 ≈4 mg of spray‐dried NG‐NPA powder was re‐suspended in 16 mL of 1× phosphate‐buffered saline (PBS) at room temperature (RT) for drug release studies. For immediate release measurement, 0.5 mL of the suspension was centrifuged using an Amicon Ultra‐0.5 mL centrifugal filter unit with a 100 kDa molecular weight cut‐off at 1500 × g for 3 min. For time‐course analysis, 0.5 mL aliquots were collected at 0 (start), 1, and 3 h post‐incubation. Each aliquot was centrifuged under the same conditions, and the filtrate was diluted 1:10 (100 µL filtrate in 900 µL DMSO) prior to quantification by high‐performance liquid chromatography (HPLC). The cumulative release profile revealed an initial burst release of ≈35%, followed by 6% additional release at 1 h and 4% more by 3 h. To assess release under shear‐induced disaggregation, 7 mL of NG‐NPA suspension was sonicated using a physiotherapy‐based sonicator (Sonicator Model 740, Mettler Electronics Corp.) at a 1 Hz pulse frequency and a total energy output of 2.2 W cm^−^^2^ for a total of 3 min, applied in 1‐min on/1‐min rest cycles. Drug release measured immediately after sonication showed a 21% release, indicating enhanced release under mechanical shear.

### Fabrication of NG‐NPAs via Spray Drying and Their Characterization—Shear Breakability Assay

An ultrasound sonication assay was used to quantify the shear‐induced disaggregation of NG‐NPAs, as previously described.^[^ ^66^ ^]^ This method provides a simple and robust means to assess the shear breakability of NPAs, consistent with previously established shear response profiles.^[^ ^29^, ^66^ ^]^ Briefly, suspensions of NG‐NPAs or Blank‐NPAs (1 mg mL^−1^ in PBS, 3 mL) were subjected to ultrasound stimulation for 3 min using a physiotherapy‐grade Sonicator 740 (Mettler Electronics Corp.) at an intensity of 2.2 W cm^−^^2^, with a 5 cm^2^ applicator and 20% pulse setting, corresponding to an approximate acoustic pressure of ≈150 kPa.^[^ ^66^ ^]^ The reported power density (2.2 W cm^−^^2^) reflects the spatial average temporal peak, while the acoustic pressure refers to the peak‐to‐peak amplitude. Quantitative analysis of particle size distributions before and after sonication was performed using laser diffraction (Mastersizer, Malvern Instruments) to evaluate shifts in mean particle size. NG‐NPAs exhibited an average reduction in mean particle size of 15 ± 3% following ultrasound exposure, which was comparable to that observed with Blank‐NPA aggregates (13 ± 2%). These results indicate that NG‐NPAs possess similar shear breakability characteristics to previously reported NPAs used for shear‐responsive drug delivery (Table S3, Supporting Information).^[^ ^29^ ^]^

### Surgical Procedures—Laser Doppler Flowmetry (LDF)

LDF was used to measure changes in cerebral blood flow (CBF) in the MCA territory. The LDF probe (Moor Instruments, UK) was inserted into the hollow PEEK screw (Solid Spot LLC, Santa Clara, CA, USA) located +5 mm lateral of midline and ‐2 mm posterior to Bregma in the right parietal bone.

### Surgical Procedures—Laser Speckle Contrast Imaging (LSCI)

LSCI (RWD Life Sciences, China), through bilateral thinned‐skull cranial windows, was used to measure changes in CBF in the distal MCA territory supplied by retrograde collateral flow on the right (stroke) side of the brain and in the matching region contralaterally. Bilateral cranial windows (5 mm x 5 mm) were created extending posterolaterally from Bregma 1 mm posterior, 1 mm lateral, according to our published method.^[^ ^22^ ^]^ Regions of interest (ROI) on laser speckle imaging software were placed 2 mm posterior and 3 mm lateral from Bregma to measure changes in CBF within the the terminal MCA perfusion territory, supplied by LMC (Figure 1a). Placement of ROI were based off cerebrovascular casting studies^[^ ^67^, ^68^ ^]^ and have been validated using magnetic resonance imaging^[^ ^69^ ^]^ and hydrogen clearance.^[^ ^70^ ^]^ A matching ROI was placed in the homotypic contralateral region. To measure cortical bridging vein diameters LSCI with higher magnification was used to zoom in on the right (stroke) side of the brain. Veins were identified as larger cortical vessels (≈100 µm in diameter) draining from the cerebral cortex to the superior sagittal sinus, according to descriptions from previous studies^[^ ^71^, ^72^ ^]^ (Figure S2, Supporting Information). The LSCI ldata was collected at pre‐stroke baseline, post‐stroke pre‐infusion, and then continuously during 40 min drug infusion. Perfusion data analysis and vein diameter analysis was performed by an investigator blind to treatment groups, using LSCI analysis software (RWD Life Sciences, China).

### Surgical Procedures—Intracranial Pressure (ICP)

An ICP probe (OpSens Fiber Optic Pressure Sensors, Canada) was inserted epidurally through a hollow PEEK screw in the left frontal bone, according to our previously described method.^[^ ^73^ ^]^ The screw was secured using ethyl 2‐cyanacrylate (Super Glue Ultra‐Fast liquid, UHU, Australia) and biocompatible caulking material (Silagum, Gunz Dental, Germany).

### Surgical Procedures—Middle Cerebral Artery Occlusion (MCAo)

MCAo was induced according to our established protocol.^[^ ^74^ ^]^ To summarize, a silicone‐tipped monofilament was inserted into the external carotid artery and advanced 20 mm up the internal carotid artery until resistance was felt and a drop in perfusion (>70%) on LDF was observed, indicating occlusion of the origin of the middle cerebral artery. The filament was retracted after 70 min to produce recanalisation.

### Exclusion Criteria

Subarachnoid haemorrhage, experimental complications and ≤ 70% drop on laser Doppler flowmetry at time of MCAo were pre‐specified exclusion criteria.

### Study design

*Study I –* In Study I the shear stress was investigated in LMC during experimental stroke in Wistar rats and SHRs. Detailed methods and collateral velocity and diameter data have previously been reported for the Wistar rats and SHRs.^[^ ^22^, ^61^ ^]^ However, the shear stress was not previously analysed. Rats underwent baseline recordings of ACA–MCA LMC blood flow velocity and diameter before induction of experimental stroke (90 and 70 min MCAo respectively, n = 6 Wistars and n = 5 SHRs). LMC flow velocity and diameter recordings were then taken every 10 min throughout occlusion. Shear stress was then calculated using blood flow velocity, diameter, and viscosity.^[^ ^23^ ^]^ For shear stress calculations see Methods in the Supporting Information. (A detailed experimental timeline can be found in Figure S3A, Supporting Information)

*Study II –* In study II the effect of NG‐NPAs was determined on LMC‐mediated penumbral perfusion during stroke in SHRs. Pre‐stroke baseline measurements of LMC perfusion, contralateral perfusion and mean arterial pressure (MAP), were taken, and then rats were subjected to 70 min of MCAo. A post‐occlusion/pre‐drug infusion baseline measurement of LMC and contralateral perfusion, and MAP was conducted 25 min after MCAo. Animals were then randomized by sealed numbered envelope to receive intravenous infusion of either Blank‐NPAs (control, 4 mg NPAs in 2 mL of saline, n = 7) or NG‐NPAs (50 µg NG in 4 mg NPAs in 2 mL of saline at 2.8‐3 mL h^−1^ = 4 µg kg min^−1^, n = 7), for 40 min. We found that Blank‐NPAs yielded similar neurological outcomes to saline treatment in stroke animals (Figure S4, Supporting Information) and thus Blank‐NPAs were used for controls in all other experiments Considering NG's short half‐life (≈4 min), an intravenous infusion was chosen as the desired route and mode of administration to allow for sustained effect of the treatment. Dose and sample sizes were based on results from a preliminary study shown in the Supporting Information. All treatments were administered by a surgeon who was blind to treatment allocation at the drug administration stage and remained blinded during all subsequent analysis of results. LMC perfusion, contralateral perfusion and MAP recordings continued until thread withdrawal (recanalization) 70 min post‐MCAo. Following 24 h of recovery, animals were tested for stroke‐induced neurological deficits and brains were collected for infarct volume, performed using both Triphenyltetrazolium (TTC) staining and haematoxylin and eosin (H&E) histology per our standard technique^[^ ^75^ ^]^ (detailed methods in Supporting Information and experimental timeline in Figure S3B, Supporting Information).

*Study III –* In study III the effect of NG‐NPAs was determined on cortical vein diameter and ICP. The experimental protocol was the same as study II, except that higher magnification of laser speckle contrast imaging was used and ICP was also measured. Laser speckle imaging was performed through the cranial window prior to induction of MCAo and during infusion of NG‐NPAs (n = 6) or Blank‐NPAs (n = 6). Changes in cortical bridging vein diameters and ICP was monitored at pre‐stroke baseline and post‐stroke at pre‐infusion baseline and throughout 40 min drug infusion. ICP data was recorded for the NG‐NPAs (n = 3) and blank‐NPAs (n = 4) groups (detailed timeline in Figure S3C, Supporting Information).

*Study IV*‐ In Study IV the effect of free nitroglycerin (free‐NG) was determined on LMC perfusion and MAP during stroke. The protocol was as for Studies II and III, except animals were randomized to receive intravenous infusion of saline (n = 6) or infusion of NG (0.25 µg µl^−1^ at 300 µl h^−1^ = 4 µg kg min^−1^ of NG, n = 6 or 0.5 µg µl^−1^ at 300 µl h^−1^ = 40 µg kg min^−1^ of NG, n = 4), commencing 25 min after MCAo (detailed timeline in Figure S3D, Supporting Information).

### Statistical Analysis

Data were processed in Microsoft Excel, and all statistical analysis was performed using GraphPad Prism 10 (GraphPad, USA). D'Agostino and Pearson omnibus normality tests were performed on all data. When data was tested for outliers, a ROUT test (Q  =  1%) was used, and outliers were removed from data sets unless they were biologically relevant. The primary outcome measure was changes in LMC perfusion in Study II. All other outcomes were secondary outcomes and considered exploratory. In our preliminary study conducted in SHR rats it was found that NG‐NPA treatment increased collateral perfusion by 42 ± 27% versus ‐3 ± 7% for the NG‐NPA group (Figure S5, Supporting Information). These values give a Cohen's d effect size of 2.25. Therefore, 7 animals per treatment group was required to be able to reject the null hypothesis that NG‐NPAs do not enhance collateral flow (primary outcome) with probability (power) 0.95. The type I error probability associated with the test of this null hypothesis (alpha) was 0.05. All other analysese of secondary endpoints had an n = 3–7 per group). When comparing two‐independent variables, normally distributed data was compared using unpaired student's *t*‐test and non‐normally distributed data was compared using Mann Whitney‐U test. When comparing how a response was affected by two factors where one of the factors was repeated; independence, normality, and sphericity was assumed, and variables were compared using Repeated measures two‐way ANOVA with Sidak's, Dunnett's or Uncorrected Fisher's LSD multiple comparisons test between groups. All comparisons were two‐tailed. Correlations were classified as tiny or < 0:05, very small (0:05 < = r < 0:1), small (0:1< = r < 0:2), medium (0:2< = r < 0:3), large (0:3< = r < 0:4), or very large (r > = 0:4) according to Funder and Ozer's criteria.^[^ ^76^ ^]^ Detailed description of sample size calculations for LMC perfusion are available in the Supporting Information. Statistical tests used for each analysis are reported in figure legends. For ANOVAs, overall ANOVA results are reported in figure legends and results of post hoc tests are reported in the results text and are represented in each figure. Significant differences were accepted at the level of *p* < 0.05; data are presented as mean ± SEM.

## Conflict of Interest

The authors DJB, DEI, NK, and OU are inventors on patents covering the NPA technology and methods for its use. AMB is a senior medical science advisor and co‐founder of Brainomix, a company that develops electronic ASPECTS (e‐ASPECTS), an automated method to evaluate ASPECTS in stroke patients. All other authors declare other no conflict of interest.

## Author Contributions

Donald E Ingber and Daniel J Beard to signficy co‐senior author. A.M.B., N.J.S., D.E.I., and D.J.B. did conceptualization. M.L., S.A., R.J.H., K.M., D.P., D.O., F.W., YKY, AC, N.H., S.J., P.G.B., F.M., C.G.E., D.B., O.U., N.K., D.J.B., and D.E.I. did methodology. M.L., N.J.S., and D.J.B. did Investigation. M.L., N.J.S., and D.J.B. did visualization. Y.C., A.M.B., N.J.S., D.E.I., and D.J.B. did Funding acquisition. D.J.B. did Project administration. N.J.S., D.J.B., and D.E.I. did Supervision. M.L. did Writing – original draft. M.L., S.A., R.J.H., K.M,. D.P., D.O., F.W., O.U., D.B., N.K., Y.C., A.M.B., N.J.S., D.J.B., and D.E.I. did Writing – review & editing.

## Supporting information



Supporting Information

## Data Availability

The data that support the findings of this study are available from the corresponding author upon reasonable request.
